# Demographic and clinical predictors of health-related quality of life among people with type 2 diabetes mellitus living in northern Thailand: A cross-sectional study

**DOI:** 10.1186/s12955-019-1246-2

**Published:** 2019-12-03

**Authors:** Saneh Khunkaew, Ritin Fernandez, Jenny Sim

**Affiliations:** 1 Boromarajonani of Nursing College Uttaradit, Praboromarajchanok Institute, 38/40 Jasadabordit Rd, Muang Uttaradit, 50300 Thailand; 2 School of Nursing, University of Wollongong, Northfields Ave, Wollongong, NSW 2522 Australia

**Keywords:** Health-related quality of life, Diabetes mellitus, Diabetic foot ulcer, Thailand, Predictor and nursing

## Abstract

**Background:**

Type 2 Diabetes Mellitus (T2DM) is a chronic disease which is growing global health problems. However, research on such prediction of health-related quality of life (HRQOL) in Thailand is limited, in particular on the demographic and clinical characteristic in each HRQOL domains. Therefore, the aim of the present study was to determine the demographic and clinical predictors of health-related quality of life among people with type 2 diabetes mellitus (T2DM) in Northern Thailand.

**Methods:**

A cross-sectional study of people with T2DM at a large teaching hospital in Northern Thailand was conducted. The HRQOL was evaluated using the Thai version of Diabetes-39. Descriptive analysis was used to summarize the demographic and HRQOL scores. Multiple regression analysis was used to determine the predictors of overall HRQOL and the predictors of each D-39 dimension.

**Results:**

A total of 502 people with T2DM were recruited. Forty-one were identified as having diabetic foot ulcers. The mean score for perception of overall HRQOL was 61.18 (SD 18.74). Scores in the D-39 questionnaire showed a poor HRQOL among people with T2DM. The predictors of demographic and clinical characteristics of people with T2DM were calculated for overall HRQOL and all six domains.

**Conclusion:**

These results demonstrate that people with T2DM have a poor HRQOL. The presence of diabetic foot ulcers and smoking status were identified as significant predictors of low HRQOL in the domains relating to diabetes control, social burden and energy and mobility presence of obesity, receiving insulin injection or a combination of insulin and oral medication were predictors of poor HRQOL in the domain of other health problems and diabetes complications. These findings allow for a nursing care plan for diabetes management to achieve optimal glycaemic control and improve their HRQOL.

## Introduction

Evidence indicates that there is an increasing prevalence of diabetes both in developed and developing countries [1]. In the United States, Selvin et al. [2] found that the prevalence of diabetes among older adults has risen from 5.8% in 1988–1994 to 12.4% in 2005–2010. Similarly, in Canada, Greiver et al. [3] estimated the prevalence of diabetes was 7.6% of the population. In Thailand the number of people with diabetes is rapidly increasing due to changing lifestyle [4] with the estimated national prevalence of diabetes reported to be 9.6% (2.4 million people) [5].

Living with diabetes has a significant impact on the Health-Related Quality of Life (HRQOL) of those affected [6]. The evidence demonstrates that people with diabetes have a poor quality of life particularly in physical and psychological functions [7] compared to those with no chronic illness [8, 9]. Various demographic factors impact on the HRQOL of people with T2DM. While some studies suggest that males have a lower general health condition, physical function, and physical role limitation, others report contradictory findings [10, 11]. Age also influences the HRQOL of people with diabetes, with older people having poorer HRQOL compared to younger people [12–14]. Income levels have also been reported to impact the HRQOL of people with T2DM [15, 16]. Similary, the length of time a person has had T2DM influences HRQOL with longer periods resulting in lower HRQOL [13]. People with T2DM who smoke have also been reported to have poorer HRQOL compared to non smokers [17].

There are a range of clinical characteristics that impact on the HRQOL of people with T2DM. The use of insulin and / or oral anti diabetic medications have been identified as predictors of poor HRQOL among people with T2DM [18]. The cross-sectional study among Hong Kong Chinese adults with T2DM reported that BMI was negatively associated with the physical component summary (PCS-12) [19]. Presence of diabetic foot ulcers (DFUs) has also been reported to have a negative effect on several domains of a person’s HRQOL including daily and social activities [20]. Results from a recent systematic review that included 11 studies of people with DFUs identified that the HRQOL of participants in most of the studies was poor, particularly in physical functioning, role physical, general health, and vitality [21]. Furthermore, people experiencing pain due to a DFU have an even lower HRQOL [22]. In addition, people with abnormal biomedical indicators including Glycosylated Haemoglobin (HbA1c) have also been reported to have poorer HRQOL [23].

While there is a plethora of research on the HRQOL among people with diabetes living in developed countries there is limited published literature in developing countries such as Thailand, despite the rapidly increasing prevalence of T2DM in that country. Therefore, the aim of this study was to determine the HRQOL and predictors of HRQOL in people with T2DM who are living in Northern Thailand. This will help to inform strategies to improve HRQOL among people with T2DM and reduce the incidence of diabetes complications.

## Methods

This study used a descriptive, cross-sectional design to determine demographic and clinical predictors of HRQOL among people attending the diabetes outpatient clinic at a large teaching hospital in Northern Thailand. This study is part of a larger study assessing the HRQOL, diabetes knowledge and self-care management among Thai people with T2DM. Recruitment commenced on 13th September and was completed on 13th November 2016.

### Sample

A consecutive sampling strategy was used to recruit participants. People were included in the study if they were: more than 18 years old; diagnosed with T2DM; willing to participate; and able to read or understand the Thai language. People who had cognitive impairment or communication difficulties were excluded.

### Data collection

All eligible potential participants were informed about the study by a research assistant using a standardised script in plain Thai language. People were also advised that participation in the study was voluntary and that non-participation would not affect the care they received at the hospital. Informed consent was obtained from those who met the inclusion criteria and participants were asked if they wished to complete the survey themselves or if they wished to complete the survey using the interview method. Those who wished to complete the survey themselves were given a copy of the questionnaire and were asked to place the completed questionnaire in a secure box at the diabetic clinic. For those willing to participate using the interview method, a registered nurse conducted a 1–1 interview and obtained the data. The four registered nurses who assisted with data collection participated in a half-day workshop that included data collection techniques and a mock data collection trial supervised by the lead researcher. HRQOL was assessed using the Thai version of the Diabetes-39 questionnaire.

### Data collection instruments

Data were collected relating to participant demographics, clinical characteristics and HRQOL. The demographic data collected included: gender; age; smoking status; marital status; education level; employment status; income; and occupation. The clinical characteristics data obtained were; length of time since diagnosis with T2DM; diabetes therapy; most recent glycosylated haemoglobin (HbA1c); Body Mass Index (BMI); and presence of a DFU.

There are numerous instruments used to measure the HRQOL in Thai people with T2DM, however the majority of these are generic and not disease specific instruments [7, 17, 24–26]. A systematic review revealed the need for the use of a validated disease specific instrument to measure HRQOL in patients with T2DM [27]. Hence, the Health-related quality of life (HRQOL) was assessed using the Thai version of the Diabetes-39 questionnaire. The Thai version of the Diabetes-39 questionnaire assesses six distinct dimensions of diabetes related to HRQOL: diabetes control (13 items); anxiety and worry (4 items); social burden (6 items); sexual functioning (3 items); energy and mobility (10 items); and other health problems and diabetes complications (3 items) [28]. Each item is rated on a seven point Likert scale, ranging from “not affected at all” to “extremely affected” [28]. The D-39 questionnaire also included an overall evaluation (2 items), which are self-perceived overall rating of HRQOL and self-perceived rating of severity of diabetes [28]. Overall HRQOL and Overall Severity of T2DM were included as individual items and assessed on a seven point Likert scale ranging from “highest quality” to “lowest quality” and “not severe at all” to “extremely severe” [28]. Permission to use the D-39 questionnaire (English and Thai version) was granted by the instrument developers.

The overall reliability of this scale has been reported to be greater than 0.7 [28]. Reliability for each dimension includes; energy and mobility (0.94); diabetes control (0.94); anxiety and worry (0.89); social burden (0.76); sexual functioning (0.88); and other health problems and diabetes complications (0.83) [28].

## Statistical analysis

All data were entered into Survey Monkey© and exported into the SPSS software version 21 (SPSS Inc., Chicago, IL, USA). The scores for each dimension, Overall HRQOL (1 item) and Overall Severity of T2DM (1 item) were transformed into 0 to 100 scales according to author guidelines [28]. The score closer to 0 indicates a better HRQOL and score closer to 100 a worse HRQOL [28]. Descriptive analysis was used to summarise demographic and HRQOL score. Univariate analysis was conducted to assess the relationship between each demographic and clinical variable on the HRQOL. Only the demographic and clinical variables that were significant in the univariate analysis were included in a standard multiple linear regression analysis to determine the predictor of HRQOL. The following demographic predictor variables were included in the regression model (a) Gender (b) age (c) marital status (d) education level (e) working status (f) income. Education level was recoded into binary variables; primary education and lower, and secondary and higher. The following clinical characteristic predictor variables were included in the regression model (a) smoking status (b) diabetes duration (c) HbA1c and (d) BMI. The beta values and confidence intervals (95%) were calculated in the multiple linear regression analyses. Statistical significance was set at *p* < 0.05.

## Results

### Participant characteristics

Of the 502 participants in the study, the majority were female (*n* = 305, 60.75%). The mean age of the participants was 60.17 ± 10.70. The majority of the participants (*n* = 366) were living with a partner. The majority of participants were educated at elementary school level (*n* = 331). A third of the participants were employed and the majority (72.70%) earned 0–10,000 baht/month. (Table 1).

**Table 1:** Demographic data (*n* = 502)

Variables	Frequency (*n* = 502)
Age (mean ± SD)	60.17 ± 10.70
Gender	
Female	305
Smoker	30
Marital Status	
Living with partner	366
Highest Qualification	
Elementary school (Primary school)	331
Secondary school (High school)	79
Diploma and over	84
Employment Status	
Employed	349
Earnings per month	
0–10,000 Baht/month	365
More than 10,001 Baht/month	132
Occupation	
Farmer	94
Government worker	22
Housewives/husbands	160
Private employee	27
Business	64
Diabetes therapy	
Insulin	32
Oral medication	318
Combination of insulin and oral medication	143
Non pharmacologic treatment	8
Clinical characteristics	Mean (SD)
Diabetes duration (years)	9.87 ± 8.13
HbA1c (mg %)	7.78 ± 1.77
BMI	26.96 ± 5.57
Presence of DFUs (n)	41

Abbreviation: DFU, diabetic foot ulcers; HbA1c, Glycosylated Haemoglobin A1c; BMI, Body Mass Index

The mean duration of diabetes was 9.87 (SD 8.13) years. The mean glycosylated haemoglobin (HbA1c) level for participants was 7.78% (61.5 mmol/mol) (SD 1.77) and the mean BMI was 26.96 kg/m^2^ (SD 5.57). Of the 502 participants 41 were identified as having DFUs.

## Health-related quality of life

The mean score for the single item summarising participants overall HRQOL was 61.18 (SD 18.74) and the single item summarising participants overall severity of T2DM was 28.45 (SD 20.56). The mean scores for each subscale were: diabetes control 19.78(SD 14.80); anxiety and worry 23.52 (SD 17.71); social burden 16.58 (SD 12.40); sexual functioning 15.89 (SD 19.28); energy and mobility 21.60 (SD 15.85); and other health problems and diabetes complications 21.43(SD 18.41). (Table 2).

**Table 2:** HRQOL among participants (n = 502)

D-39 dimension^a^	Mean (SD)
Diabetes control (13 items)	19.78 ± 14.80
Sexual functioning (3 items)	15.89 ± 19.28
Social burden (6 items)	16.58 ± 12.40
Anxiety and worry (4 items)	23.52 ± 17.71
Energy and mobility (10 items)	21.60 ± 15.85
Other health problems and diabetes complications (3 items)	21.43 ± 18.41
Overall evaluation	Mean (SD)
Self-perceived overall HRQOL (1 item)^a^	61.18 ± 18.74
Self-perceived overall severity (1 item)^b^	28.45 ± 20.56

Abbreviation: DFU, diabetic foot ulcers

^a^High score indicated poor HRQOL

^b^High score indicated severity of disease

## Demographic and clinical characteristic predictors of HRQOL

### Overall HRQOL

A multiple regression was performed for prediction of participants’ overall rating of HRQOL. The following variables that were significant in the univariate analysis were included in the prediction model: education levels, income, and use of insulin only. The multiple correlation coefficient (*R* = 0.14) was significantly different from zero, *F* = (3495) = 3.52, *p* < 0.05 and accounted for 2% of the variance in the dependent variable, as explained by the set of independent variables (*R*^*2*^ = 0.021, *R*^*2*^_*adj*_ = 0.015). None of the three variables that were significant in the univariate analysis were found to be significant predictors of overall HRQOL.

### Overall severity of T2DM

A multiple regression was performed for prediction of participants’ rating of overall severity of their T2DM. The following variables that were significant in the univariate analysis were included in the prediction model: use of insulin only and use of combination of insulin and oral medication. The multiple correlation coefficient (*R* = 0.21) was significantly different from zero, *F* = (2501) = 11.753, *p* < 0.05 and accounted for 4% of the variance in the dependent variable, as explained by the set of independent variables (*R*^*2*^ = 0.045, *R*^*2*^_*adj*_ = 0.041). Both use of insulin or combination of insulin and oral medication were found to be significant predictors of participants’ rating of the overall severity of their diabetes.

### Diabetes control

A multiple regression was performed for prediction of HRQOL in the diabetes control domain (13 items) for people with diabetes. The following variables that were significant in the univariate analysis were included in the prediction model: age, presence or absence of DFU, duration of diabetes, use of insulin only, use of a combination of insulin and oral medications, and smoking status. The multiple correlation coefficient (*R* = 0.28) was significantly different from zero, *F* = (6487) = 6.69, *p* < 0.05 and accounted for 6% of the variance in the dependent variable as explained by the set of independent variables (*R*^*2*^ = 0.076, *R*^*2*^_*adj*_ = 0.065). Younger age, longer duration of diabetes, smoking and those with DFUs had significantly worse HRQOL relating in the diabetes control domain.

### Sexual functioning

A multiple regression was performed for prediction of HRQOL related to sexual functioning (6 items). The following variables that were significant in the univariate analysis were included in the prediction model: gender, education levels and smoking status. The multiple correlation coefficient (*R* = 0.37) was significantly different from zero, *F* = (3502) = 27.68, *p* < 0.05 and accounted for 14% of the variance in the dependent variable, as explained by the set of independent variables (*R*^*2*^ = 0.142, *R*^*2*^_*adj*_ = 0.137). Non-smoking status and female gender were found to be significant predictors of better HRQOL relating to sexual functioning.

### Social burden

A multiple regression was performed for prediction of HRQOL in the social burden domain (6 items) among people with diabetes. The following variables that were significant in the univariate analysis were included in the prediction model: presence or absence of DFU, income, duration of diabetes, use of a combination of insulin and oral medications, and smoking status. The multiple correlation coefficient (*R* = 0.22) was significantly different from zero, *F* = (5487) = 5.16, *p* < 0.05 and accounted for 5% of the variance in the dependent variable, as explained by the set of independent variables (*R*^*2*^ = 0.05, *R*^*2*^_*adj*_ = 0.041). Shorter duration of diabetes, non-smoking status, and absence of DFUs were found to be significant predictors of better HRQOL relating to social burden.

### Anxiety and worry

A multiple regression was performed for prediction of HRQOL in the anxiety and worry domain (4 items) for people with diabetes. The following variables that were significant in the univariate analysis were included in the prediction model: presence or absence of DFU, income, use of a combination of insulin and oral medications, and smoking status. The multiple correlation coefficient (*R* = 0.22) was significantly different from zero, *F* = (4491) = 6.81, *p* < 0.05 and accounted for 5% of the variance in the dependent variable, as explained by the set of independent variables (*R*^*2*^ = 0.053, *R*^*2*^_*adj*_ = 0.045). Non-smoking status, and higher income levels were found to be significant predictors of better HRQOL relating to anxiety and worry.

### Energy and mobility

A multiple regression was performed for prediction of HRQOL in the energy and mobility domain (10 items) for people with diabetes. The following variables that were significant in the univariate analysis were included in the prediction model: presence or absence of DFU, income, duration of diabetes, use of insulin only, use of a combination of insulin and oral medications, and smoking status. The multiple correlation coefficient (*R* = 0.31) was significantly different from zero, *F* = (6486) = 8.58, *p* < 0.05 and accounted for 9% of the variance in the dependent variable, as explained by the set of independent variables (*R*^*2*^ = 0.096, *R*^*2*^_*adj*_ = 0.085). Shorter duration of diabetes, non-smoking status, absence of DFUs and non-use of insulin were found to be significant predictors of better HRQOL relating to energy and mobility.

### Other health problems and diabetes complication

A multiple regression was performed for prediction of HRQOL in the other health problems and diabetes complication domain (3 items) among people with diabetes. The following variables that were significant in the univariate analysis were included in the prediction model: presence or absence of DFU, income, duration of diabetes, use of insulin only, use of a combination of insulin and oral medications, and BMI. The multiple correlation coefficient (*R* = 0.28) was significantly different from zero, *F* = (6485) = 6.68, *p* < 0.05 and accounted for 7% of the variance in the dependent variable, as explained by the set of independent variables (*R*^*2*^ = 0.078, *R*^*2*^_*adj*_ = 0.066). Absence of DFUs, non-use of insulin and/or combination of insulin and oral medication and decreased BMI were found to be significant predictors of better HRQOL relating to other health problems.

## Discussion

This cross-sectional study has contributed new knowledge related to the HRQOL and in particular has identified the predictors of HRQOL among people with T2DM in Northern Thailand. In our study, the clinical characteristics of the participants including the mean duration of diabetes, glycosylated haemoglobin (HbA1c) level and BMI were similar to that reported in the extant literature [6, 28, 29].

However, in our study, participants had worse HRQOL in the domains relating to energy and mobility and other health problems and diabetes complications when compared to another study undertaken in the Thai population [28]. This could be due to the fact that our study was undertaken in Northern Thailand compared to the other study using the Thai version of the D-39 [28] which was undertaken in Southern Thailand. The lifestyles in these two regions are markedly different. Thailand is located in Southeast Asia, bordered by Laos on the North and East, Myanmar on the Northwest and west and Malaysia to the South [30] and these geographical features contribute to the cultural differences relating to religious beliefs, lifestyle, and foods that may have influenced the HRQOL.

In this study, the results obtained from the self-perceived HRQOL and disease severity mean score were 61.18 and 28.45. This result is consistent with the literature where studies have reported that people with T2DM do not perceive the relationship between HRQOL and severity of diabetes [31, 32]. This is because people value their HRQOL but do not consider their diabetes to be severe. This discrepancy requires prompt education strategies to be implemented.

In this study age was a predictor of HRQOL in the domain relating to diabetes control with increasing age resulting in better HRQOL. This result is inconsistent with the literature where studies have reported that people with T2DM aged over 40 years have poorer HRQOL [13, 14]. Our finding may relate to people who are aged less than 40 years having the ability to take care of themselves. Further research should be undertaken in the older age group for a better understanding of why older Thai people had better rating for the diabetes control domain.

Previous studies that investigated the gender differences in HRQOL using other instruments identified females with T2DM having worse HRQOL [7, 17, 24]. This is contradictory to our results where the female gender was found to be a significant predictor of better HRQOL in relation to the domain of sexual functioning. These results are consistent to those published on the same instrument survey, which shows that women were perceived to have better HRQOL [33]. This appears to be because women are more active in self-care and preventive care; seeking up to date information and therefore adapting to their diagnosis [34]. In contrast, men may be less concerned about their health conditions and this impact upon sexual activities more than women. Therefore, identifying strategies to improve HRQOL among Thai males with T2DM is important. Low income was a predictor in the anxiety and worry domain of HRQOL which is consistent with prior studies by Alfian [15] and Mngomezulu and Yang [16]. Those with high income may have more choice and be able to access higher quality medical care than people with a lower income.

This study found the presence of a DFU was a predictor of worse HRQOL in the domains relating to diabetes control, social burden, anxiety and worry, energy and mobility and other health problems and diabetes complications which is consistent with the literature [7, 9, 35]. People with DFUs incur nerve damage due to neuropathy and decreased peripheral circulation [36] which can result in severe pain which impairs their mobility and physical functioning. This may be because having a chronic wound can have a bad odour and large dressings, which can cause problems in a person’s social life and therefore anxiety and depression. Our findings have provided additional information to support health care professionals to understand the impact that body perception, hygiene and culture can have on HRQOL. We would suggest that a nursing intervention should be implemented and focused on these domains for improving HRQOL among people with T2DM.

Treatment with insulin and combination of insulin therapy and oral medication was associated with worse HRQOL in the domain of other health problems and diabetes complications and in perception of overall severity of diabetes (Table 3). This finding is consistent with previous studies [28]. Maddigan et al. [18] also reported worse HRQOL among people with T2DM who received insulin therapy or oral medication. Similarly, findings from the Dutch Diacourse study, indicated worse diabetes-related distress among people receiving insulin therapy [37].. Receiving this medication is an indication of poor glycaemic control and may indicate development of other co-morbidities such as heart disease, stroke and kidney disease which impact on vision, dexterity, ambulation, emotion and pain or discomfort which impair HRQOL.

**Table 3:** Demographic and clinical characteristic predictors of Health-related quality of life

Model	Demographic and clinical characteristic predictors			
Diabetes control		Coeff.	95% CI	Sig.
	R^2^ = 0.76; Adj R^2^ = 0.65)			
	(Constant)	56.595	40.818, 72.373	.000
	Age	−.224	−.354, −.094	.001
	Duration of diabetes	.208	.021, .395	.029
	Insulin only	5.341	−.136, 10.819	.056
	Combination of insulin and oral	2.825	−.332, 5.983	.079
	Smoking	−8.392	−13.806, −2.977	.002
	Presence of DFUs	−5.267	−10.009, −.525	.030
Sexual functioning	R^2^ = 0.14; Adj R^2^ = 0.13)			
	(Constant)	49.643	35.377, 63.908	.000
	Smoking	−9.229	−.335, .027	.008
	Gender	−12.124	−15.667, −8.582	.000
	Education level	2.748	−.853, 6.350	.134
Social burden	R^2^ = 0.05; Adj R^2^ = 0.41)			
	(Constant)	36.653	24.661, 48.645	.000
	Presence of DFUs	−4.272	−8.219, −.324	.034
	Income	−.853	−2.494, .788	.308
	Duration of diabetes	.167	.023, .311	.023
	Combination of insulin and oral	1.718	−.866, 4.303	.192
	Smoking	−6.529	−11.152, −1.906	.006
Anxiety and worry	R^2^ = 0.05; Adj R^2^ = 0.045)			
	(Constant)	58.556	41.819, 75.292	.000
	Presence of DFUs	−5.226	−10.809, .357	.066
	Income	−2.913	−5.219, −.607	.013
	Combination of insulin and oral	3.414	−.016, 6.845	.051
	Smoking	−11.195	−17.634, −4.756	.001
Energy and mobility	R^2^ = 0.096; Adj R^2^ = 0.085)			
	(Constant)	44.599	29.598, 59.599	.000
	Presence of DFUs	−5.792	−10.720, −.863	.021
	Income	−1.930	−3.986, 3.020	.066
	Duration of diabetes	.237	.053, .421	.012
	Combination of insulin and oral	3.255	−.075, 6.586	.055
	Smoking	−6.660	−12.425, −.896	.024
Other Health problems and diabetes complication	R^2^ = 0.078; Adj R^2^ = 0.066)			
	(Constant)	26.689	−4.148, 3.962	.000
	Presence of DFUs	−8.143	−.338, .033	.006
	Income	−.792	−3.203, 1.619	.519
	Duration of diabetes	2.387	−.082, .349	.224
	Insulin only	11.853	5.120, 18.585	.001
	Combination of insulin and oral	5.133	1.244, 9.022	.010
	BMI	.300	.016, .584	.038
Overall HRQOL	R^2^ = 0.021; Adj R^2^ = 0.015)			
	(Constant)	4.389	4.030, 4.748	.000
	Education level	.186	−.098, .470	.200
	Income	.127	−.071,	.207
	Insulin only	−.430	−.902, .042	.074
Overall severity	R^2^ = 0.045; Adj R^2^ = 0.041)			
	(Constant)	2.271	2.118, 2.424	.000
	Insulin only	.713	.200, 1.227	.007
	Combination of insulin and oral	.621	.344, .898	.000

The low rate of insulin use in this study is also comparable with other studies that have demonstrated that patients with T2DM reject the use of insulin [38]. This could be because they are either afraid of needles or injections, or because they erroneously think that insulin is the cause of complications.

These results indicate that evidence-based strategies need to be implemented to improve the overall HRQOL for adults with T2DM in Northern Thailand. It is a challenge for health care providers to keep a wide range of factors in mind when establishing a nursing care intervention for people with T2DM. It is important to consider which factors affect HRQOL, particularly in different regions within Thailand. This approach would attempt to holistically improve physical, mental, social and spiritual needs as well as improving glycaemic control leading to better HRQOL.

### Strength and limitations

The major strength of this study was the use of the Thai version of the Diabetes-39 which is a valid and reliable instrument for assessing the HRQOL among diabetic patients [39], compared to other studies which have used generic questionnaires for evaluating HRQOL [7, 10, 40, 41]. Another strength was that the survey was able to be completed using the interview method. This meant that all eligible participants could complete the survey regardless of literacy levels. Thirdly, all interviewers were trained in the administration of the questionnaire which added to the robustness of the research methods. Despite the strengths of this study some of the limitations inherent in undertaking such a study need to be acknowledged. The study was undertaken using a non-random sample and was conducted at a specialist diabetic clinic which could influence the results. Further, large scale multi-centre studies need to be undertaken to investigate the HRQOL of people with T2DM in the various regions of Thailand. For future research a larger sample size and the use of a disease specific questionnaire is suggested.

## Conclusions

The results of this study demonstrate that Thai people with T2DM have a poor HRQOL. None of the demographic or clinical characteristics are predictors of individual perceptions of overall HRQOL. However, in the domain of diabetes control, social burden, energy and mobility, and other health problems and diabetes complication domains, it was found that the presence of DFUs are potentially impacted by these dimensions. People with T2DM could not perform household chores and were unable to do what they wanted to do, as well as being unable to take care of daily activities. In these domains relating to diabetes control, sexual functioning, social burden, anxiety and worry and energy mobility, people who smoked had a significantly poorer HRQOL. People who were treated with insulin injection and a combination of insulin and oral medication tended to have poor HRQOL in the domain of other health problems and diabetes complication. Also, people with obesity had significantly poor HRQOL in this domain.

People with T2DM showed that their self-perceived HRQOL was poor. However, they do not consider diabetes to be a serious disease. Therefore, they do not perceive the relationship between HRQOL and disease severity. However, given the negative impact on the physical and psychological functioning of those affected with T2DM, there is an urgent need for evidence-based strategies to be implemented to prevent the complications of T2DM.

## Data Availability

The dataset used and/or analysed during the current study are available from the corresponding author on request.

## Abbreviations

BMI: Body Mass Index; HbA1c: Glycosylated Haemoglobin
D-39: Diabetes-39
DFUs: Diabetic Foot Ulcers
HRQOL: Health-Related Quality of Life
PCS-12: Physical Component Summary
T2DM: Type 2 Diabetes Mellitus
